# Supplementary material for: CriSNPr, a single interface for the curated and de novo design of gRNAs for CRISPR diagnostics using diverse Cas systems
Source: eLife. 2023 Feb 8;12:e77976. doi: 10.7554/eLife.77976 (PMC9940907; doi:10.7554/eLife.77976)
Supplement: Figure 5—source data 1. [file elife-77976-fig5-data1.docx]

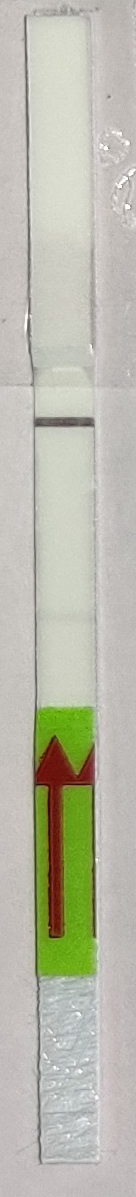

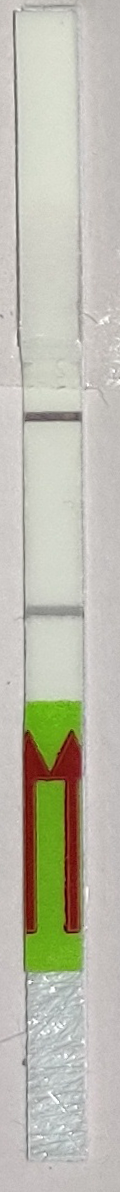


77.19 7.81 TOPSE

E484K

WT.

Source Data-2, Figure 5. The red rectangle denotes the approximate area cropped for generating Figure 5.
