# Supplementary material for: CriSNPr, a single interface for the curated and de novo design of gRNAs for CRISPR diagnostics using diverse Cas systems
Source: eLife. 2023 Feb 8;12:e77976. doi: 10.7554/eLife.77976 (PMC9940907; doi:10.7554/eLife.77976)
Supplement: Figure 5—figure supplement 2—source data 1. [file elife-77976-fig5-figsupp2-data1.zip › Figure 5ΓÇöfigure supplement 2ΓÇösource data 1.docx]

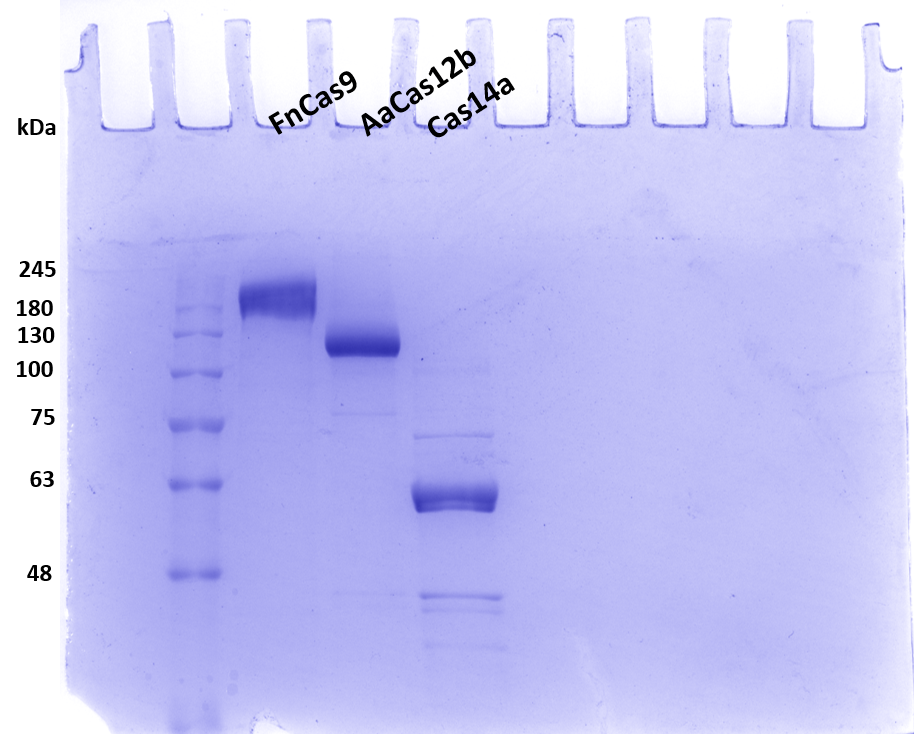


Source Data-1, Figure 5—figure supplement 2. The red rectangle denotes the approximate area cropped for generating Figure 5—figure supplement 2.
