# Supplementary material for: CriSNPr, a single interface for the curated and de novo design of gRNAs for CRISPR diagnostics using diverse Cas systems
Source: eLife. 2023 Feb 8;12:e77976. doi: 10.7554/eLife.77976 (PMC9940907; doi:10.7554/eLife.77976)
Supplement: Figure 6—source data 1. [file elife-77976-fig6-data1.docx]

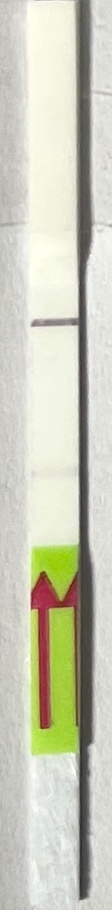

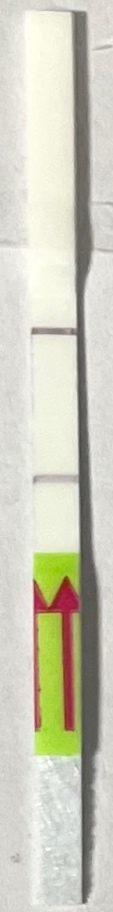

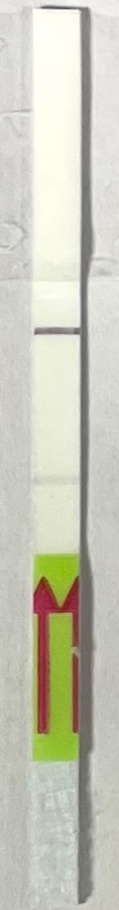

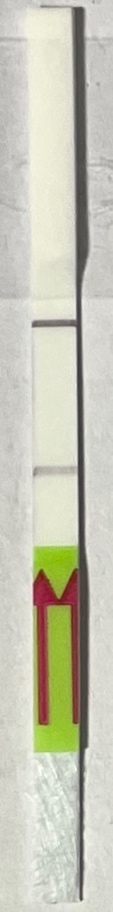

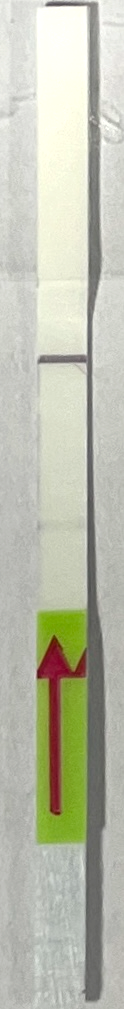

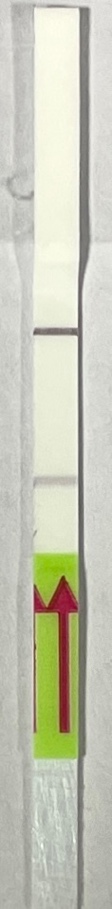


rs2073874_2&6

WT.

rs2073874_16&19

WT.

rs138739292_2&6

WT.

1.24 39.47. 8.82 38.72 2.81 34.45 TOPSE

Source Data-3, Figure 6a. The red rectangle denotes the approximate area cropped for generating Figure 6a.
